# A clinical perspective on muscle specific kinase antibody positive myasthenia gravis

**DOI:** 10.3389/fimmu.2024.1502480

**Published:** 2024-12-05

**Authors:** Omar Keritam, Angela Vincent, Fritz Zimprich, Hakan Cetin

**Affiliations:** ^1^ Department of Neurology, Medical University of Vienna, Vienna, Austria; ^2^ Comprehensive Center for Clinical Neurosciences & Mental Health, Medical University of Vienna, Vienna, Austria; ^3^ Nuffield Department of Clinical Neurosciences, University of Oxford, Oxford, United Kingdom

**Keywords:** muscle-specific kinase, myasthenia gravis, MuSK-MG, IgG4, neuromuscular junction, autoimmune disorder, review

## Abstract

The discovery of autoantibodies directed against muscle-specific kinase (MuSK) in “seronegative” myasthenia gravis (MG) patients marked a milestone in MG research. In healthy muscle, MuSK regulates a phosphorylation pathway, which is essential for the development and maintenance of acetylcholine receptor (AChR) clusters at the neuromuscular junction. Autoantibodies directed against MuSK are predominantly of the IgG4 subclass, but there is increasing evidence that IgG1-3 could also contribute to the pathology underlying MuSK-MG. MuSK-IgG4 are monovalent and block the binding site for LRP4 on MuSK, thereby inhibiting the downstream phosphorylation pathway and compromising the formation of AChR clusters. Clinically, MuSK-MG is commonly associated with the predominant involvement of bulbar, facial, shoulder and neck muscles. Cholinesterase inhibitors should be avoided in MuSK-MG due to the risk of clinical impairment and cholinergic crisis. Corticosteroids and other non-steroidal immunosuppressants are less effective with the need for higher doses and prolonged treatment. Rituximab, by contrast, has been shown to be particularly effective and is now often used early in the disease course. Its use is associated with a significant improvement in the clinical outcome of MuSK-MG patients over time. This review aims to describe the pathophysiology underlying MuSK-MG and provide a comprehensive overview of the clinical features and therapeutic options.

## 1 Introduction

The first known documentation of myasthenia gravis (MG) by Thomas Willis dates back to the second half of the seventeenth century, and around 200 years later W.H. Erb (1879) and S. Goldflam (1893) published the first detailed descriptions of the disease (1–3). In 1895, Jolly first theorized that an impairment of signal transmission at the neuromuscular junction (NMJ) was the underlying pathomechanism (4), but it was not until the 1970s that the acetylcholine receptor (AChR) antibody (Ab) was identified (5). In the year 2001, Hoch et al. followed with the publication of their discovery of Abs against muscle specific kinase (MuSK) (6). Both discoveries were significant milestones: the identification of AChR-Abs proved the proposition of John Simpson in 1960 that MG was an autoimmune disease (7), and the discovery of MuSK-Abs confirmed hints of clinical distinctions between “AChR positive” and previously “seronegative” MG.

MG with underlying MuSK-Abs (MuSK-MG) is characterized by specific clinical features and a distinct treatment response when compared to patients with AChR-Abs (8). Muscle weakness and fatigability primarily affect bulbar, respiratory and neck muscles (9–14). Atrophy of affected muscle groups was observed in later stages of the disease, although imaging studies have also revealed early subclinical muscular wasting (12, 14–19). Before the identification of MuSK-Abs, the treatment of “seronegative” MG patients followed the standard regimen for AChR-Ab positive MG (AChR-MG). However, part of that regimen, namely thymectomy and cholinesterase inhibitors, was ineffective in MuSK-MG (20–22). Cholinesterase inhibitors were even associated with the potential to worsen symptoms of MuSK-MG patients (10, 12, 22, 23). Advancements in our understanding of the molecular mechanisms underlying MuSK-MG have been successfully translated into clinical practice and led to improved treatment strategies. Clinical guidelines on MG now take the antibody status into consideration (24, 25). Different recommendations for AChR-MG and MuSK-MG have been implemented, respectively, which has led to a significant improvement of the clinical outcome of MuSK-MG patients over time (25, 26). In light of these developments, this review aims to address our understanding of the pathomechanisms involved in MuSK-MG, the clinical features, as well as current and future therapeutic approaches.

## 2 MuSK is a crucial component of the AChR clustering pathway

Muscle specific tyrosine kinase is a 120 kDa transmembrane protein located in the postsynaptic muscle cell membrane and plays a crucial role in the development, function, and maintenance of the NMJ (27–29). Structurally, MuSK is composed of three Ig-like domains followed by a frizzled-like cysteine-rich domain in the N-terminal extracellular regions, a transmembrane region, and a cytoplasmic domain with kinase activity (30–33).

### 2.1 The agrin/LRP4/MuSK/Dok7 pathway as a positive regulator of AChR clustering

**Figure 1:**
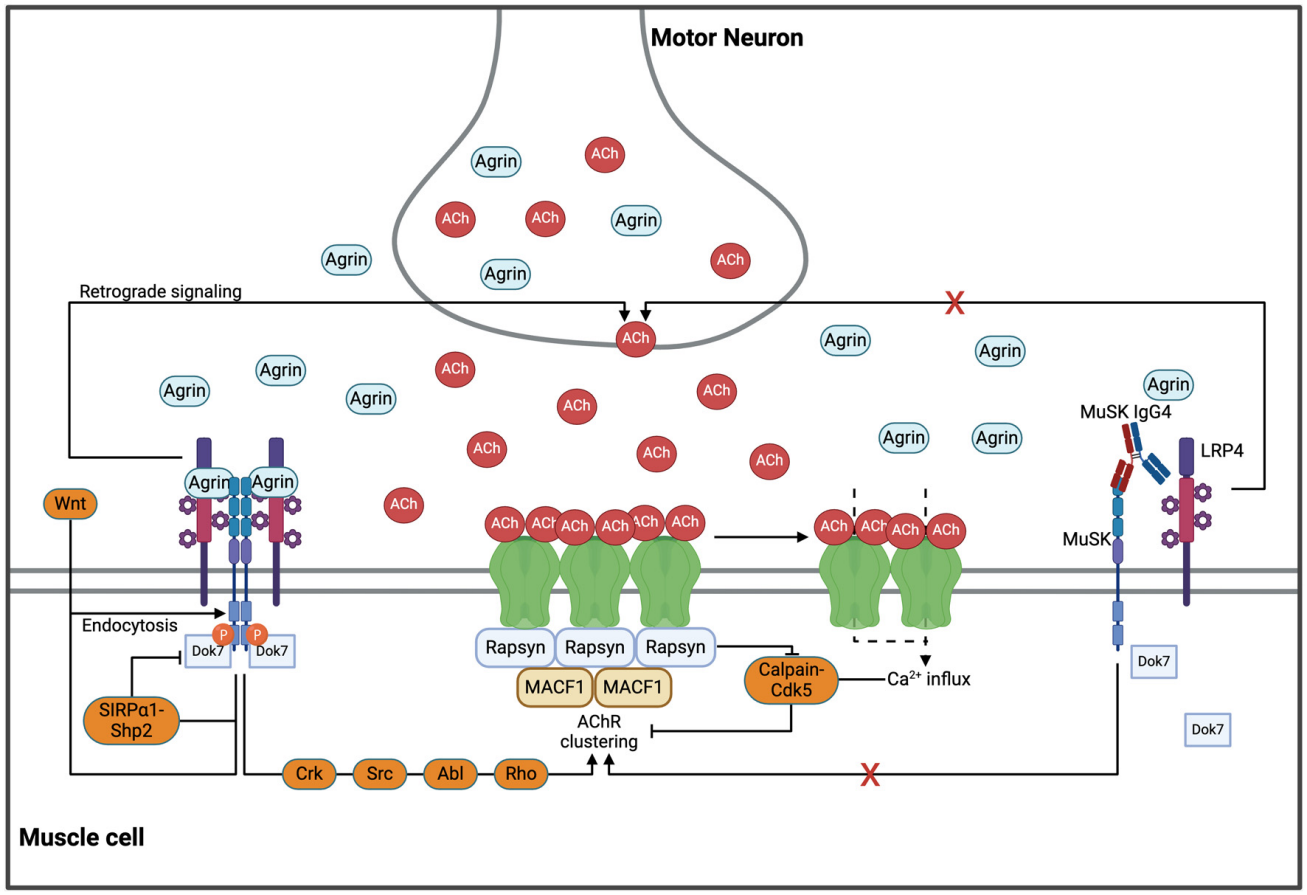
The AChR clustering pathways in health and MuSK-MG. MuSK, agrin and LRP4 bind in a 2:2:2 configuration, facilitating transmembrane autophosphorylation of MuSK. Recruitment of Dok7 initiates a signaling cascade through various pathways (including Src, Crk, Abl and Rho) which in turn enhances rapsyn-condensation and subsequent AChR clustering. The two-dimensional AChR network is connected through rapsyn, which forms up to three bridges with all AChR subunits. This network is further stabilized in the third dimension by the cytoskeleton via MACF1. Formation and stabilization of AChR clusters is essential for physiological neuromuscular activity. However, excessive cholinergic activation leads to a prolonged Ca^2+^ influx through the AChRs, thereby activating the Calpain/Cdk5 pathway and dispersing AChR clusters. Further upstream, MuSK-dimerization is inhibited by the SIRP a 1/SHP2 pathway in a negative feedback loop. Endocytosis of MuSK, either mediated by agrin or Wnts, is suggested as another important negative regulator. LRP4-MuSK is hypothesized to increase the quantal content, which balances AChR clustering. However, the exact mechanisms underlying this effect are yet to be unraveled. Monovalent IgG4 antibodies inhibit MuSK dimerization and the downstream (and upstream) pathways important for AChR clustering. Abl, Abelson murine leukemia viral oncogene homolog 1; ACh, acetylcholine; AChR, acetylcholine receptor; Cdk5, Cyclin-dependent kinase 5; Crk, CT10 regulator of kinase; Dok7, Downstream of tyrosine kinase 7; LRP4, low-density lipoprotein receptor-related protein 4; MACF1, microtubule actin crosslinking factor 1; MuSK, muscle specific kinase; Rho, Ras homology; SHP2, Src homology region 2 domain-containing phosphatase-2; SIRP a 1, signal regulatory protein α1; Src, from “sarcoma”, Wnt, wingless-related integration site. Created in BioRender.com.

Neural agrin, a large heparan-sulfate proteoglycan secreted from the motor nerve terminal, and the low-density lipoprotein receptor-related protein 4 (LRP4), another transmembrane protein of the muscle cell membrane, interact with MuSK molecules in a 2:2:2 configuration (34–39). In a recently published study analyzing the structure of the agrin/LRP4/MuSK complex using cryogenic electron microscopy, LRP4 was shown to act as a “clamp” resulting in a direct interaction between agrin and MuSK, thereby promoting MuSK dimerization and (auto)phosphorylation of its cytoplasmic domain (37). Downstream of tyrosine kinase 7 (Dok7) is recruited and binds to the juxtamembrane cytoplasmic region of MuSK. Upon phosphorylation, Dok7 fosters full MuSK activation by enhancing MuSK dimerization (40). Subsequently, MuSK activation induces tyrosine-phosphorylation of 43 kDa receptor-associated protein of the synapse (rapsyn), which then undergoes liquid-liquid phase separation (LLPS) to form membraneless condensates (41). These co-condense with AChR subunits and cytoskeletal proteins, facilitating the formation of rapsyn-AChR-aggregates together with components of the cytoskeleton (41). Different intermediate proteins including Crk, Abl, Src and Rho are recruited by MuSK activation and further amplify the process of LLPS and AChR clustering (42–49). Ultimately, rapsyn interacts with the cytoplasmic loops of all AChR subunits and connects receptors by up to three bridges, which is the minimum number required to form a 2D network (50–52). Many of these pathways are involved in actin remodeling and cytoskeletal rearrangements to anchor and immobilize the AChR clusters within the postsynaptic plasma membrane (52, 53) (**Figure 1**).

### 2.2 Negative regulators of AChR clustering

It is important to acknowledge that AChR clustering underlies a homeostatic regulation of positive and negative regulators. As highlighted before, the reciprocal activation of MuSK and Dok7 is the strongest promoter of AChR clustering. Acetylcholine (ACh), by contrast, is a strong negative regulator. Sustained cholinergic stimulation associated with prolonged calcium influx through the AChRs (e.g., in the absence of acetylcholine esterase in the synaptic cleft) with accumulation of intracellular calcium activates a calpain-Cdk5 pathway, which mediates AChR dispersal (54–58). This pathway is counterbalanced by rapsyn, which was reported to interact with calpain and inhibit its activity, thereby stabilizing AChR clusters in an agrin-dependent manner (54). Another negative regulator of AChR clustering is the SRC homology two domain-containing phosphotyrosine phosphatase 2 (SHP2). Agrin-stimulation of MuSK was shown to phosphorylate signal regulatory protein a 1 (SIRP a 1), an activator of SHP2, which, in a negative feedback loop, reduces MuSK phosphorylation and activity (59). Endocytosis of MuSK was suggested to be another important regulator of AChR clustering and is directly linked to its activation, specifically in response to stimulation with agrin (60, 61). Other factors promoting MuSK endocytosis include Wnt, a family of glycoproteins secreted from the nerve terminal that regulate AChR clustering and play a critical role in neuromuscular development (62). MuSK also acts as a receptor for the bone morphogenic protein (BMP). The MuSK-BMP pathway has been suggested to inhibit Ca^2+^ signaling via regulator of G protein signaling 4 (RGS4) (63). Together, all these mechanisms constitute a complex network of pathways that contribute to the fine-tuned regulation of AChR clustering and cholinergic signaling at the NMJ (**Figure 1**).

## 3 Unravelling the pathogenicity of MuSK antibodies

The pathogenicity of MuSK-Abs is related to the nature of the antibodies, which are predominantly of the IgG4 subclass and therefore unable to bind complement or activate immune cells via interaction with Fcγ receptors (64, 65). Instead, IgG4 is considered to mediate anti-inflammatory tolerance effects; in general, by competitive blocking of epitopes on autoantigens, IgG4 can prevent the binding of other antibody classes and neutralize their effector mechanisms (64). In the case of MuSK-MG, the pathogenicity of MuSK-IgG4-Abs relies on their ability to block the MuSK-LRP4 interaction and prevent MuSK activation, which disturbs the balance between MuSK-induced clustering and ACh-induced dispersal of AChR clusters (66, 67).

### 3.1 MuSK antibodies are monovalent and undergo Fab-arm exchange

MuSK-IgG4-Abs are, unlike IgG1-3 antibodies, functionally monovalent. Due to variations in the amino acid sequence (Ser228), IgG4 are characterized by a greater stereometric flexibility in the hinge region, which causes an instability of the interchain disulfide bridges, and the alternative formation of more stable intrachain bridges under reducing conditions (68–70). As a result, IgG4-Abs dissociate into two half-molecules, each consisting of a heavy and a light chain, which then recombine randomly into chimeric, bi-specific antibodies (64, 68, 71). This mechanism is further facilitated by another single amino acid change in the CH3 region (Arg409) that reduces the non-covalent interaction between both heavy chains. Taken together, these amino acid changes allow for ~99% of MuSK-IgG4-Abs to undergo Fab-arm exchange (71, 72), which are therefore monovalent for their antigen.

### 3.2 MuSK-IgG4 antibody inhibition of LRP4-MuSK signaling and complex interactions with both pre- and post-synaptic proteins

The reactivity of MuSK-Abs can be directed against several extracellular MuSK domains (64, 73), with epitope spreading reported in 19% of MuSK-MG patients (73). Disease severity, however, is determined by reactivity of the autoantibodies against the MuSK-Ig-like domain 1 (MuSK-Ig1) (66, 67, 73, 74), which is required for MuSK to bind LRP4 (38). MuSK-IgG4-Abs block the interaction between MuSK and LRP4 and prevent MuSK phosphorylation and activation (**Figure 1**). As a result, MuSK becomes unable to respond to agrin-LRP4 stimulation, and the agrin-LRP4-MuSK-Dok7 cascade does not form, resulting in reduced AChR clustering at the NMJ (66, 67). The disrupted LRP4-MuSK interaction was also shown to correlate with a lack of the compensatory presynaptic increase of released acetylcholine vesicles (i.e., quantal content, QC) in *in vivo* models of MuSK-MG, which, by contrast, is commonly observed in AChR-MG disease models and in patient muscles (75–77). A potential explanation is based on the function of LRP4 as a retrograde signal molecule (78, 79). LRP4-MuSK is suggested to increase the QC, which would balance the LRP4-MuSK induced AChR clustering by increasing AChR-dispersal. MuSK-Abs could then disrupt this mechanism and prevent the compensatory increase of the quantal content, although this has not been shown directly so far (76, 77, 80).

The loss of acetylcholinesterase (AChE) has also been proposed as a pathomechanism in MuSK-MG. MuSK interacts with collagen Q (ColQ), which is arranged as trimers to form a triple helix in the synaptic cleft, where each ColQ molecule binds and anchors a tetramer of AChE to the basal lamina (81, 82). The MuSK-Ig1 and -Fz domains have been determined as the binding sites for ColQ (74) with MuSK-Abs shown to block these interaction sites and inhibit the binding of MuSK to ColQ (83). This could lead to the loss of AChE from the extracellular matrix of the NMJ and increase the availability of acetylcholine in the synaptic cleft, which, as a negative regulator of AChR clustering, could facilitate dispersal of the receptors. This was hypothesized to be responsible for the hypersensitivity of many MuSK-MG patients towards AChE inhibitors, although there was no correlation between drug hypersensitivity and autoantibodies against specific epitopes in a longitudinal epitope mapping study in MuSK-MG (73).

### 3.3 MuSK-IgG1-3 antibodies could contribute to the pathogenic process in some MuSK-MG patients

MuSK-IgG1-3 can be detected at low concentrations in most MuSK-MG patients and have been proposed to contribute to the pathogenic process in MuSK-MG (67, 84). They were shown to inhibit agrin-induced AChR clustering in C2C12 cells, surprisingly without affecting LRP4-MuSK interaction. Both IgG1-3 and IgG4 prevented AChR cluster formation in C2C12 cells or in C2C12 cells overexpressing Dok7, which induces dimerization and activation of MuSK and thereby facilitates AChR clustering independent of the agrin-LRP4 pathway (67). In a more recent study, MuSK IgG1-3 induced a similar phosphorylation time course for MuSK, Dok7 and the AChR β-subunit as agrin but still prevented the formation of AChR clusters induced by agrin in C2C12 cells (84). Further evidence for the potential pathogenic role of MuSK IgG1-3 antibodies derives from experiments utilizing monoclonal antibodies isolated from MuSK-MG patient B cell clones (85–87). Here, even in the absence of agrin, divalent antibodies recognized the MuSK-Ig-like domain 2 and enhanced MuSK phosphorylation but inhibited AChR clustering, irrespective of the subclass backbone (86). In another study, divalent MuSK-Abs were found to target the MuSK Ig-like 1 domain and again resulted in increased MuSK phosphorylation. However, instead of inhibiting the clustering of AChRs, these antibodies induced low levels of AChR clustering, which was explained by the ability of divalent antibodies to crosslink MuSK molecules (85). These findings could indicate different downstream MuSK phosphorylation and AChR clustering pathway aspects depending on the IgG subclass or on the different MuSK epitopes targeted by the monoclonal or polyclonal (patient serum) antibodies. This hypothesis is supported by experiments analyzing MuSK mutations identified in patients with congenital myasthenic syndromes. Here, a mutation in the MuSK Fz-like domain ablated MuSK phosphorylation and AChR clustering, whereas another mutation in the kinase domain of *MUSK* was found to increase MuSK phosphorylation while impairing AChR clustering (88). Therefore, the modification of different MuSK domains or epitopes by mutations or antibodies could differentially affect the conformation and activation of MuSK and, thereby, also the downstream signaling pathway required for AChR clustering (87, 89, 90).

The activation of complement could represent another mechanism underlying MuSK IgG1-3 pathogenicity, with evidence deriving from *in vivo* disease models. MuSK-immunized mice show elevated levels of complement-fixing anti-MuSK IgG isotypes in sera and NMJs, and complement deposition can be observed widely in muscle samples (91, 92). Moreover, MuSK-immunized IgG1 knock-out mice (where mouse IgG1 is the murine analogue of human IgG4 with poor complement activation potency) and WT both developed comparable disease severities, serum MuSK-Ab levels, and NMJ immunoglobulin and complement deposition ratios (91). However, *in vivo* experiments performed by another group revealed the induction of experimental autoimmune MG in mice by the passive transfer of MuSK-IgG4 but not MuSK-IgG1-3 together with complement (93), although the MuSK-specific IgG1-3 antibodies could not be detected in the mouse sera. It was therefore suggested that this model was not representative of the situation in MuSK-MG patients, in which MuSK-IgG1-3 antibodies are commonly detectable (67, 93).

## 4 Epidemiology of MuSK-MG

MuSK-Abs can be detected in up to 70% of generalized MG patients without AChR-Abs (6, 10), and LRP4-Abs can be found in 7-33% of double-seronegative patients without AChR- and MuSK-Abs (94). Double positive patients with MuSK- and LRP4-Abs were found more frequently than double positive patients with AChR- and LRP4-Abs (14.9% versus 7.5%) (94). The overall female-to-male ratio of MuSK-MG ranges between 3:1 and 9:1 (10, 12, 13), with higher rates of male MG patients in late-onset disease (9, 95). In contrast to AChR-MG, a bimodal pattern of incidence is not common in MuSK-MG (10), with only one study from China reporting a bimodal distribution of disease onset (9). Prevalence ratios vary significantly across countries (**Table 1**), with differences between ethnic groups. In a Norwegian and Dutch study, MuSK-MG was more frequent in Asian immigrants than in patients of European descent (8% vs. 0-4%) (96). In an US-American study, 50% of patients with MuSK-MG were African-American, as compared to only 10% within the AChR-MG cohort (97). Interestingly, different rates of MuSK-MG were reported within individual countries. In a Chinese study, only 4% of AChR-Ab negative patients in the HuBei province had MuSK-Abs (98), while another study reported a rate of 26.7% among AChR-Ab negative patients in Northeast China (99). However, these differences are likely due to the use of different assays in the two studies, with cell-based assays resulting in a lower proportion of MuSK-Ab positives compared to enzyme-linked immunosorbent assays. A particularly high prevalence (43.0 per 1,000,000 population) of MuSK-MG was reported in Sardinia (100). The same study reported a much higher overall incidence and prevalence of MG in Sardinia compared to other regions/countries (96, 100–104). Notably, Sardinia is burdened by particularly high frequencies of other autoimmune disorders, such as multiple sclerosis (MS) and systemic lupus erythematosus (SLE) (105, 106). The *TNFSF13B* variant, resulting in an overexpression of the B cell activating factor (BAFF), was found to be associated with MS and SLE in a genome-wide association study in Sardinia (107) and can help to explain the higher frequencies of myasthenia gravis. Differences in the frequencies of specific HLA alleles across populations could also explain the geographical variations. A meta-analysis published in 2018 concluded that DQ5-DR14 and DQ5-DR16 haplotypes are associated with an increased risk of developing MuSK-MG, whereas DQ3-DR4 and DQ3-DR11 were found to be protective (108).

**Table 1:** Prevalence of MuSK-MG *.

Region	Publication year	Prevalence (per million)
China, HuBei Province (98)	2007	0.02**
Netherlands (101)	2007	1.9
Greece (102)	2009	2.9
Italy, Trento (173)	2011	1.9
Northern Europe (Norway & Netherlands) (96)	2016	3.6
Northern Portugal (103)	2016	5.8
Latvia (174)	2017	8.9
Italy, Sardinia (100)	2024	43.0

* Only epidemiological studies with available prevalence estimations or with figures that permitted prevalence calculations were included.

** Another study from Northeast China reported higher rates of MuSK-MG (99); this study, however, did not permit prevalence calculations and was thus not included in the table.

## 5 MuSK-MG patients present with distinct clinical features

MuSK-MG patients are characterized by specific clinical symptoms that differ from those of MG patients with AChR-Abs. An acute or subacute onset of extraocular or bulbar muscle weakness is common, with bulbar muscles often remaining severely affected throughout the disease (9–13, 95). In contrast to AChR-MG, extraocular muscle paresis is mostly conjugated in MuSK-MG, manifesting as symmetrical upward or horizontal gaze limitation and, in some cases, progressing to complete ophthalmoplegia (109). In patients with a purely ocular onset, symptoms usually generalize within 1-3 months (109). Neck muscles are also often affected resulting in head drop (11, 13), whereas neck flexors can only be mildly involved (8, 110). Limb muscle affection is less severe and inconsistent (10, 12), and fluctuations of myasthenic symptoms may be less evident than in AChR-Ab positive MG patients (8). Myasthenic crises, by contrast, are frequent and often occur early in the disease course (9, 12, 13). Compared to LRP4-MG, MuSK-MG has been associated with a more severe clinical phenotype at disease onset, with MuSK/LRP4-Abs double positive patients being more severely affected than single positive MuSK-MG patients (94). Muscle atrophy and fatty replacement have been reported to occur frequently in chronic MuSK-MG patients, often affecting facial and bulbar muscles (16). Subclinical changes may even be detected at early disease stages by magnetic resonance imaging (111).

## 6 Diagnostic considerations in MuSK-MG

Diagnosis of MuSK-MG is based on the clinical presentation together with electrophysiological studies and the detection of MuSK-IgG-Abs. Repetitive nerve stimulation (RNS) and single-fiber jitter recording are helpful in supporting the diagnosis of MG but must be applied in affected muscles in order to prevent false-negative results in MuSK-Ab positive MG, with the sensitivity of RNS reported to increase up to 70-80% when tested in facial muscles and ranging around 20% in (unaffected) limb muscles (97, 112–114). Edrophonium/neostigmine testing is also commonly performed in MuSK-MG patients to enhance neuromuscular transmission by increasing the synaptic concentration of acetylcholine, and was shown to be positive in up to 60-70% of tested MuSK-MG patients (10, 12), which is generally lower than in AChR-Ab positive MG patients (115). However, edrophonium/neostigmine testing should be performed with caution as nicotinic side effects are common (23, 116).

Three different assays are available for the detection of MuSK-IgG-Abs, which represents the gold standard for confirmation of MuSK-MG diagnosis in suspected cases. The radioimmunoprecipitation assay (RIPA) utilizes immunoprecipitation of the extracellular domain of ^125^I-radiolabelled MuSK upon incubation with patients’ sera (117). This method is characterized by a specificity of almost 100% (118) and allows the quantification of autoantibody titers by counting the ^125^I precipitation at different serum concentrations. However, the strict regulations of radiation disposal limit its application. As a non-radioactive method, enzyme-linked immunosorbent assays (ELISA) are commercially available, and their application do not require specific licenses for diagnostic laboratories. MuSK-ELISA plates are coated with the extracellular domain of MuSK and allow quantification of MuSK-IgG titers, but the sensitivity and specificity of the assay were shown to be suboptimal compared to RIPA and cell-based assays (CBAs) (71, 119), indicating potential limitations in their diagnostic accuracy for MuSK-MG. Cell-based assays, by contrast, are characterized by higher diagnostic accuracy compared to RIPA and ELISA (120). Here, HEK293 cells are commonly used to express full-length MuSK with appropriate posttranslational modifications and conformation in the more physiological environment on the cell surface. In contrast to fixed cells, the application of live cells in CBAs was shown to be associated with higher sensitivity (121), and the use of IgG-specific secondary antibodies is recommended to avoid detection of non-specific binding of IgM to MuSK, which can be a problem with anti-human IgG antibodies that include anti-IgM activity (122). Ideally, CBAs should be used as a first-line assay in the diagnosis of MuSK-MG, as well as in the evaluation of patients tested negative by RIPA or ELISA (120).

## 7 Current therapeutic options in MuSK-MG

**Figure 2:**
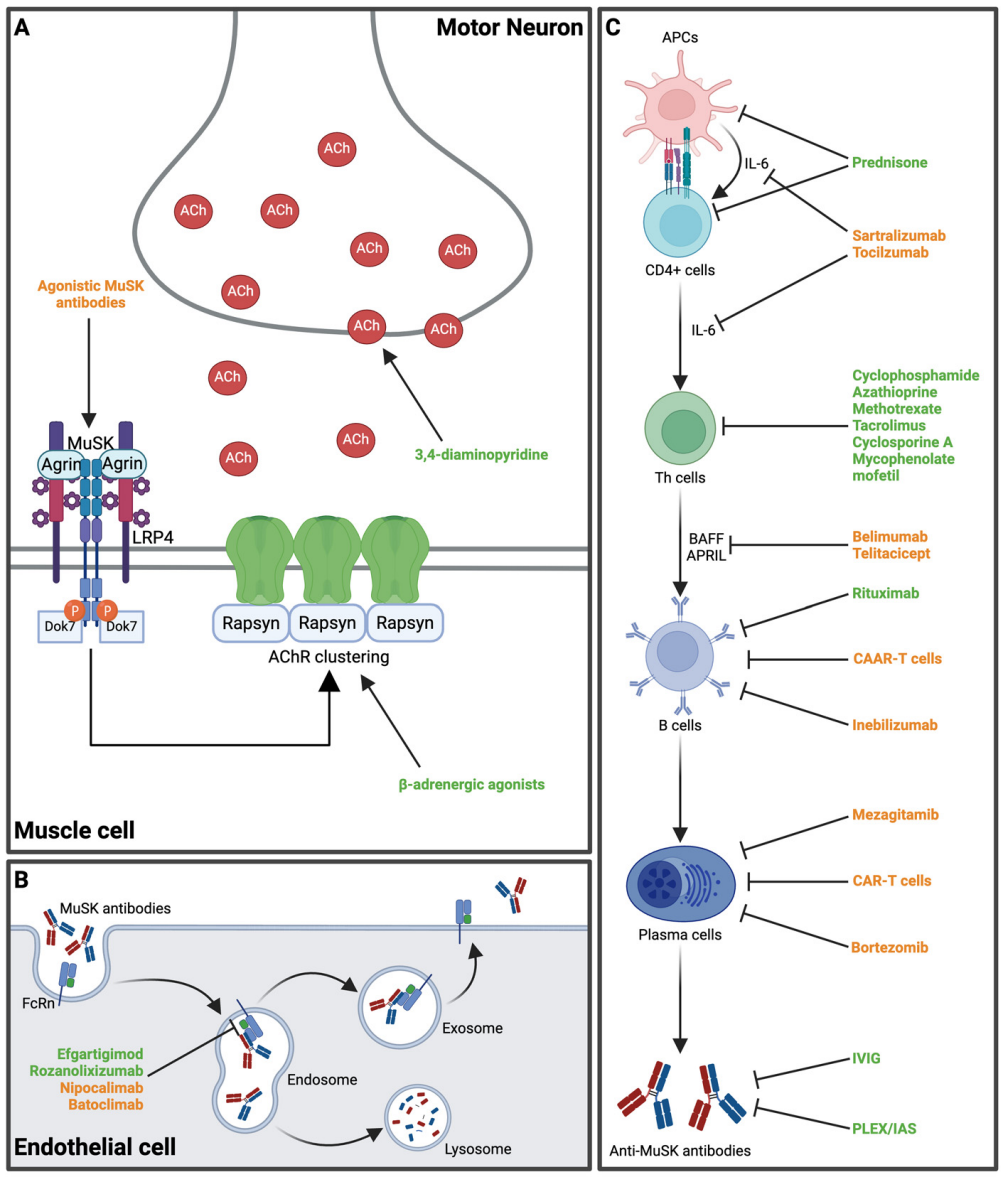
Main mechanisms of action of several established and novel therapeutic approaches in MuSK-MG. **(A)** At the neuromuscular endplate, 3,4-diaminopyridine increases pre-synaptic acetylcholine release. β-adrenergic agonists (e.g., salbutamol) stabilize AChR-clustering and increase shape and size of post-synaptic folds. Agonistic MuSK-antibodies may induce MuSK-dimerization and have been shown to reduce disease activity in a preclinical mouse model. **(B)** The FcRn are expressed on endothelial cells and mediate recycling of MuSK-Abs, thereby extending their half-life. FcRn inhibitors block the IgG-FcRn interaction and expedite the clearance of MuSK-Abs. **(C)** The pathway from antigen presentation to naïve T cells and subsequent antibody production presents different target points for therapeutic interventions. Various novel therapeutics target B cell activation and B cell lineages (see also **Table 2**). Available treatment options are highlighted in green, while novel therapeutic agents proposed for MuSK-MG are highlighted in orange. ACh, acetylcholine; AChR, acetylcholine receptor; APCs, antigen-presenting cells; APRIL, A proliferation-inducing ligand; BAFF, B cell activating factor; CAAR, chimeric autoantibody receptor; CAR, chimeric antigen receptor; CD, cluster of differentiation; Dok7, Downstream of tyrosine kinase 7; FcRn, neonatal fragment crystallizable receptor; IAS, immunoadsorption; IL-6, interleukin-6; IVIG, intravenous immunoglobulin; LRP4, low-density lipoprotein receptor-related protein 4; MuSK, muscle specific kinase; PLEX, plasma exchange; Th cells, T helper cells. Created in BioRender.com.

Specific considerations have to be taken into account for the successful treatment of MuSK-MG, owing to its distinct pathophysiological mechanisms that differ from those of MG with AChR-Abs (**Figure 2**). Gaining insight into these pathomechanisms has helped to improve the clinical management of MuSK-MG, which has evolved from an AChR-antibody negative, severe, and difficult-to-treat disease to a MuSK-antibody positive condition with a beneficial outcome in most cases. The excellent treatment response of MuSK-MG to rituximab is one major factor in this development (26).

**Table 2:** Clinical studies on novel therapeutic agents for the treatment of MuSK-MG.

Target	Name	Study identifier and phase
CD19 (B cells)	Inebilizumab (MEDI-551)	NCT04524273, phase III
CD38 (Plasma cells, B & T cells)	Mezagitamib (TAK-079)	NCT04159805, phase II
IL-6	Sartralizumab Tocilizumab	NCT04963270, phase III NCT05067348, phase II
BAFF	Belimumab Telitacicept	NCT01480596, phase II NCT04302103, phase II
B cell maturation antigen (BCMA)	CAR-T cells	NCT05451212, phase IIb
B cells expressing MuSK	MuSK-CAAR-T	NCT05451212, phase I
FcRn	Batoclimab Nipocalimab	NCT05403541, phase III NCT04951622, phase III

### 7.1 Acetylcholinesterase inhibitors

Acetylcholinesterase inhibitors (AChEI) are often ineffective and associated with an increased risk of adverse events in MuSK-MG (10, 12, 23). In a retrospective cohort analysis of 165 MuSK-MG patients receiving AChEI, only 4% reported an initial clinical improvement, whereas 34% reported an impairment of muscle weakness. Side effects were experienced by 77% of patients, with cholinergic crises occurring in 7% of the cohort (23). Impaired tolerance against AChEI could be explained by an increased sensitivity to cholinergic agents or an inherent cholinergic hyperactivity in MuSK-MG patients. Electrophysiological studies revealed a higher frequency of repetitive compound action potentials in MuSK-MG patients using AChEI (116, 123), which correlated with poor tolerance to pyridostigmine (123). These clinical data are in line with data from MuSK-immunized mice that display neuromuscular hyperactivity upon administration of AChEI (124). In another study, pyridostigmine treatment potentiated the MuSK-IgG-induced decline in AChR density and endplate potential (EPP) amplitude, whereas treatment with 3,4-diaminopyridine elevated EPP amplitude without exacerbating the MuSK-IgG-induced loss of AChRs (125). Some patients have also been reported to carry a polymorphism in the promoter region of the *ACHE* gene that encodes the catalytic subunit of AChE associated with increased susceptibility to adverse events upon (even low dose) AChEI treatment (126).

### 7.2 3,4-diaminopyridine

3,4-diaminopyrdine improves neuromuscular transmission by increasing the presynaptic release of acetylcholine. Evidence for effectiveness in MuSK-MG derives from animal models (125), case reports (127, 128), and a randomized-controlled study on 7 patients (129). Here, treatment with 3,4-diaminopyrdine in a daily dose of 30-60mg was associated with improvement of disease severity according to different clinical scores including the Quantitative MG score and the MG-specific Activities of Daily Living score. However, as the number of patients was very low in this study, there is a need for confirmation of the results in a large multi-center trial.

### 7.3 β-adrenergic agonists

Sympathomimetics have been shown to maintain NMJ structure by increasing the shape and size of the postsynaptic folds (130, 131), and to improve NMJ function (131). The β-adrenergic agonists salbutamol and ephedrine have been well established for the therapy of congenital myasthenic syndromes with *AGRN*, *COLQ*, *DOK7*, *LRP4* and *MUSK* mutations (132, 133), but with only anecdotal clinical experience of salbutamol in MuSK-MG (134, 135). When tested in 5 patients who had not tolerated pyridostigmine, there was a notable clinical response in 3 patients, whereas treatment had to be discontinued in 2 patients due to side effects or lack of efficacy after six months (134).

### 7.4 Corticosteroids and non-steroidal immunosuppressants

Immunosuppression is the mainstay treatment in both AChR-MG and MuSK-MG. Despite the lack of randomized-controlled trials, there is general consensus that MuSK-MG patients respond to corticosteroids and/or non-steroidal immunosuppressants (13, 136, 137), which are recommended and often used as the first-line therapy in MuSK-MG (24, 25). However, the frequency of exacerbations during corticosteroid tapering is notably higher in patients with MuSK-MG compared to those with AChR-MG, often requiring higher dosages and prolonged treatment durations (136, 138, 139). Non-steroidal immunosuppressants such as azathioprine, mycophenolate mofetil, cyclosporine, methotrexate and tacrolimus are therefore often introduced early in the disease course as steroid-sparing agents to avoid side effects associated with long-term administration of high-dose corticosteroids (13, 136, 139). However, a significant proportion of MuSK-MG patients on high-dose immunosuppression with corticosteroids alone or in combination with azathioprine have a refractory disease (136, 139). Periodic relapses and myasthenic crises can be effectively treated with acute plasma exchange or the administration of intravenous immunoglobulins (IVIG) (25), with evidence for higher rates of clinical improvement associated with plasma exchange as compared to IVIG treatment (10, 13, 140).

### 7.5 Anti-CD20 monoclonal antibodies

Rituximab is a mouse/human monoclonal chimeric IgG1 targeting CD20, a transmembrane phosphoprotein expressed on the surface of developing B lymphocytes but not on progenitor cells and mature plasma cells (141). Upon binding to CD20, immune effector cells are recruited and cause B cell lysis and depletion. Rituximab has been shown to be particularly effective in IgG4 related diseases (142–144). In MuSK-MG, there is currently class IV evidence for a beneficial effect upon treatment with rituximab deriving from a prospective blinded multicenter study (145). Especially in patients with high disease activity or refractory disease, rituximab treatment should therefore be considered early in the disease course (24, 25), which together with corticosteroids and immunosuppression results in an overall favorable outcome in most MuSK-MG patients (26). The intravenous application of rituximab is generally well tolerated and safe (146), with a rapid and sustained effect (147, 148) that correlates with a reduction of MuSK-IgG4 titers, but not with total IgG4 titers (148, 149). This may be related to MuSK-Ab production by short-lived Ab-secreting plasmablasts derived from CD20^+^ memory B cells (86, 150), in contrast to AChR-MG, where autoantibody production is maintained by long-lived plasma cells (151, 152). Relapse after treatment with rituximab was shown to correlate with the occurrence of antibody-secreting CD27^+^ CD38^+^ plasmablasts (150), and CD27^+^ memory B cell and MuSK-Ab titer monitoring were proposed as a sensitive biomarker for prediction of clinical relapses and for adjustment of rituximab infusion frequencies (150, 153). Moreover, MuSK-Ab secreting B cell clones were shown to survive B cell depletion therapy and recirculate up to several months prior to clinical relapse (154). These clones could differentiate from CD20^low^ plasmablasts (154), and their survival might be explained by the ineffectiveness of rituximab to reach tissue-based B cells (155–157).

### 7.6 FcRn blockade

The neonatal Fc receptor (FcRn) binds to the Fc region in a pH-dependent manner and protects IgG from lysosomal degradation, allowing it to return to the circulation. FcRn inhibitors such as efgartigimod and rozanolixizumab prevent FcRn from interacting with IgG, thereby disrupting the recycling process and leading to increased degradation and clearance of IgG from the circulation (158). In MuSK-MG, there is both preclinical and clinical evidence for the therapeutic potential of FcRn inhibition. Efgartigimod-treated MuSK-myasthenic mice functionally outperformed control mice, and electromyography demonstrated improved decrement and compound muscle action potentials upon treatment, which correlated with reduced MuSK-IgG4 levels (159). Efgartigimod has been tested in the phase III ADAPT trial including 129 AChR-MG and 38 AChR-Ab negative MG patients, among which 6 patients were MuSK-Ab positive and 3 patients received efgartigimod (160). However, all MuSK-MG patients responded to the therapy as defined by a ≥2 point improvement in the MG Activities of Daily Living (MG-ADL) score, including those in the placebo-group. Rozanolixizumab has also been tested in a phase III trial (MycarinG) including 21 MuSK-MG patients, with 13 patients receiving rozanolixizumab and 8 patients in the placebo group (161). All 12 MuSK-MG patients in the treatment group with available data were MG-ADL responders, in contrast to only one of seven responding patients in the placebo group. Despite the low number of MuSK-Ab positive patients included in these clinical trials, the available data suggest that FcRn inhibitors pose a promising therapeutic option for MuSK-MG and can be considered in refractory patients with unsatisfactory response to rituximab (25).

## 8 Future therapies

Despite the availability of various treatment options, a significant proportion of MuSK-MG patients remain symptomatic or experience periodic clinical exacerbations. Therefore, there is an urgent need for novel therapeutic strategies targeting the underlying immune machinery that produces the autoantibodies (162).

Given the pivotal role of B cell pathophysiology in MuSK-MG, as highlighted by the efficacy of rituximab, expanding therapeutic options to include agents that directly or indirectly target B cell lineages may provide valuable treatment alternatives. Inebilizumab (MEDI-551), an anti-CD19 monoclonal antibody, is currently under investigation in a phase III trial (MINT trial, NCT04524273). Mezagitamib (TAK-079), targeting CD38 expressed on plasma cells, completed a phase II trial in 2023 (results pending, NCT04159805). Targeting IL-6 to inhibit B cell proliferation and activation, the monoclonal antibody satralizumab (phase III LUMINESCE trial completed in 2024, preliminary results published (163), NCT04963270) and tocilizumab (ongoing phase II trial, NCT05067348) may also have therapeutic potential in MuSK-MG. Preliminary results from the LUMINESCE trial indicate significant improvement in clinical scores in generalized MG (163). However, only 9 MuSK-MG patients were included, and they were not considered in the analyses. Telitacicept (RC18), an inhibitor of the B cell activation factor (BAFF), demonstrated efficacy in reducing clinical severity in generalized MG in a phase II study (164), but this study was not able to recruit MuSK-MG patients. Similarly, in a phase II trial on the BAFF inhibitor belimumab, the primary endpoint was unmet, and MuSK-MG patients were limited to the placebo arm (165). Bortezomib, a proteasome complex inhibitor, was successfully used in a single case with MuSK-MG (166).

A novel approach utilizes chimeric antigen receptor (CAR) T cells that were genetically engineered to target the B cell maturation antigen and cause mature plasma cells to undergo apoptosis. In a prospective, open-label phase 1b/2a proof-of-concept study (MG-001), the safety and efficacy of this approach was investigated in 14 patients with generalized MG, of whom 2 patients were MuSK-Ab positive (167). Both patients improved in all clinical scales, without any dose-limiting toxicity, neurotoxicity, or cytokine release syndrome across the whole cohort. A similar approach was tested in an experimental autoimmune MuSK-MG mouse model. T cells were engineered to express the MuSK chimeric autoantibody receptor (CAAR) with CD137-CD3ζ signaling domains for precision targeting of B cells expressing anti-MuSK-Abs (168). The results were promising and indicated MuSK-specific B cell depletion without decreasing total B cell numbers or total IgG levels. In another recently published preclinical study, agonistic MuSK antibodies were successfully utilized to treat mice with severe disease activity after passive transfer of pathogenic MuSK-Abs derived from patients with MuSK-MG (169). Overall, these innovative approaches have the potential to specifically target the underlying immune mechanisms responsible for autoantibody production or directly target the disease mechanism.

C5 complement inhibitors have demonstrated efficacy in treating generalized AChR-Ab positive MG (161, 170–172). MuSK-Abs, by contrast, are predominantly of the IgG4 subclass and do not activate complement (64); therefore, the use of complement inhibitors does not appear to be indicated in MuSK-MG. However, since MuSK-IgG1-3 can also be detected at low concentrations in most MuSK-MG patients (67, 84), and given the evidence for complement activation in animal models (91, 92), complement inhibition may also represent a potential therapeutic approach for MuSK-MG with IgG1-3, which has not yet been validated in clinical trials.

## 9 Conclusion

Due to a better understanding of the underlying pathophysiological mechanisms and due to advances in the clinical management of the disease, MuSK-MG has become a condition with a favorable outcome in most cases. However, MuSK-MG patients are underrepresented in clinical trials and comparative investigations between existing drugs are yet to be performed, rendering attempts to develop treatment algorithms challenging. The implementation of consensus guidelines for clinical trials on MuSK-MG would help to standardize research efforts, ensuring more robust and comparable data across studies. Biomarker research on refractory disease may further bolster decision making processes.

## References

[1] Goldflam S. Ueber einen scheinbar heilbaren bulbärparalytischen Symptomen complex mit Betheiligung der Extremitaten. Dtsch Z Nervenheilk. (1893) 4:312–52. doi: 10.1007/BF01665294

[2] Erb W. Zur Casuistik der bulbären Lähmungen. Archiv f Psychiatrie. (1879) 9:325–50. doi: 10.1007/BF02666475

[3] Willis T. De Anima Brutorum. Oxford: Oxonii Theatro Sheldoniano (1672) p. 404–7.

[4] Jolly F. Über myasthenia gravis pseudoparalytica. Berl Klin Wochenschr. (1895) 32:1–7.

[5] Lindstrom JM Seybold ME Lennon VA Whittingham S Duane DD. Antibody to acetylcholine receptor in myasthenia gravis. Prevalence, clinical correlates, and diagnostic value. Neurology. (1976) 26:1054–9. doi: 10.1212/WNL.26.11.1054 988512

[6] Hoch W McConville J Helms S Newsom-Davis J Melms A Vincent A. Auto-antibodies to the receptor tyrosine kinase MuSK in patients with myasthenia gravis without acetylcholine receptor antibodies. Nat Med. (2001) 7:365–8. doi: 10.1038/85520 11231638

[7] Simpson JA. Myasthenia gravis: A new hypothesis. Scottish Med J. (1960) 5:419–36. doi: 10.1177/003693306000501001

[8] Rodolico C Bonanno C Toscano A Vita G. MuSK-associated myasthenia gravis: clinical features and management. Front Neurol. (2020) 11:660. doi: 10.3389/fneur.2020.00660 32793097 PMC7390870

[9] Zhou Y Chen J Li Z Tan S Yan C Luo S. Clinical features of myasthenia gravis with antibodies to MuSK based on age at onset: A multicenter retrospective study in China. Front Neurol. (2022) 13:879261. doi: 10.3389/fneur.2022.879261 35463138 PMC9033288

[10] Guptill JT Sanders DB Evoli A. Anti-MuSK antibody myasthenia gravis: clinical findings and response to treatment in two large cohorts. Muscle Nerve. (2011) 44:36–40. doi: 10.1002/mus.22006 21674519

[11] Sanders DB El-Salem K Massey JM McConville J Vincent A. Clinical aspects of MuSK antibody positive seronegative MG. Neurology. (2003) 60:1978–80. doi: 10.1212/01.WNL.0000065882.63904.53 12821744

[12] Evoli A Tonali PA Padua L Monaco ML Scuderi F Batocchi AP. Clinical correlates with anti-MuSK antibodies in generalized seronegative myasthenia gravis. Brain. (2003) 126:2304–11. doi: 10.1093/brain/awg223 12821509

[13] Pasnoor M Wolfe GI Nations S Trivedi J Barohn RJ Herbelin L. Clinical findings in MuSK-antibody positive myasthenia gravis: a U.S. experience. Muscle Nerve. (2010) 41:370–4. doi: 10.1002/mus.21533 19882635

[14] Wolfe GI Oh SJ. Clinical phenotype of muscle-specific tyrosine kinase-antibody-positive myasthenia gravis. Ann N Y Acad Sci. (2008) 1132:71–5. doi: 10.1196/nyas.2008.1132.issue-1 18567855

[15] Zhou L McConville J Chaudhry V Adams RN Skolasky RL Vincent A. Clinical comparison of muscle-specific tyrosine kinase (MuSK) antibody-positive and -negative myasthenic patients. Muscle Nerve. (2004) 30:55–60. doi: 10.1002/mus.20069 15221879

[16] Farrugia ME Robson MD Clover L Anslow P Newsom-Davis J Kennett R. MRI and clinical studies of facial and bulbar muscle involvement in MuSK antibody-associated myasthenia gravis. Brain. (2006) 129:1481–92. doi: 10.1093/brain/awl095 16672291

[17] Farrugia ME Kennett RP Hilton-Jones D Newsom-Davis J Vincent A. Quantitative EMG of facial muscles in myasthenia patients with MuSK antibodies. Clin Neurophysiol. (2007) 118:269–77. doi: 10.1016/j.clinph.2006.10.004 17157556

[18] Vincent A Bowen J Newsom-Davis J McConville J. Seronegative generalised myasthenia gravis: clinical features, antibodies, and their targets. Lancet Neurol. (2003) 2:99–106. doi: 10.1016/S1474-4422(03)00306-5 12849266

[19] Borges LS Richman DP. Muscle-specific kinase myasthenia gravis. Front Immunol. (2020) 11:707. doi: 10.3389/fimmu.2020.00707 32457737 PMC7225350

[20] Clifford KM Hobson-Webb LD Benatar M Burns TM Barnett C Silvestri NJ. Thymectomy may not be associated with clinical improvement in MuSK myasthenia gravis. Muscle Nerve. (2019) 59:404–10. doi: 10.1002/mus.26404 30575980

[21] Ponseti JM Caritg N Gamez J Lopez-Cano M Vilallonga R Armengol M. A comparison of long-term post-thymectomy outcome of anti-AChR-positive, anti-AChR-negative and anti-MuSK-positive patients with non-thymomatous myasthenia gravis. Expert Opin Biol Ther. (2009) 9:1–8. doi: 10.1517/14712590802588831 19063688

[22] Punga AR Flink R Askmark H Stalberg EV. Cholinergic neuromuscular hyperactivity in patients with myasthenia gravis seropositive for MuSK antibody. Muscle Nerve. (2006) 34:111–5. doi: 10.1002/mus.20515 16453324

[23] Ricciardi R Latini E Guida M Koneczny I Lucchi M Maestri M. Acetylcholinesterase inhibitors are ineffective in MuSK-antibody positive myasthenia gravis: Results of a study on 202 patients. J Neurol Sci. (2024) 461:123047. doi: 10.1016/j.jns.2024.123047 38759248

[24] Narayanaswami P Sanders DB Wolfe G Benatar M Cea G Evoli A. International consensus guidance for management of myasthenia gravis: 2020 update. Neurology. (2021) 96:114–22. doi: 10.1212/WNL.0000000000011124 PMC7884987 33144515

[25] Wiendl H Abicht A Chan A Della Marina A Hagenacker T Hekmat K. Guideline for the management of myasthenic syndromes. Ther Adv Neurol Disord. (2023) 16:17562864231213240. doi: 10.1177/17562864231213240 38152089 PMC10752078

[26] Tomschik M Hilger E Rath J Mayer EM Fahrner M Cetin H. Subgroup stratification and outcome in recently diagnosed generalized myasthenia gravis. Neurology. (2020) 95:e1426–e36. doi: 10.1212/WNL.0000000000010209 32641537

[27] Glass DJ Apel ED Shah S Bowen DC DeChiara TM Stitt TN. Kinase domain of the muscle-specific receptor tyrosine kinase (MuSK) is sufficient for phosphorylation but not clustering of acetylcholine receptors: required role for the MuSK ectodomain? Proc Natl Acad Sci U S A. (1997) 94:8848–53. doi: 10.1073/pnas.94.16.8848 PMC23162 9238066

[28] Burden SJ Yumoto N Zhang W. The role of MuSK in synapse formation and neuromuscular disease. Cold Spring Harb Perspect Biol. (2013) 5:a009167. doi: 10.1101/cshperspect.a009167 23637281 PMC3632064

[29] Valenzuela DM Stitt TN DiStefano PS Rojas E Mattsson K Compton DL. Receptor tyrosine kinase specific for the skeletal muscle lineage: expression in embryonic muscle, at the neuromuscular junction, and after injury. Neuron. (1995) 15:573–84. doi: 10.1016/0896-6273(95)90146-9 7546737

[30] Jennings CG Dyer SM Burden SJ. Muscle-specific trk-related receptor with a kringle domain defines a distinct class of receptor tyrosine kinases. Proc Natl Acad Sci U S A. (1993) 90:2895–9. doi: 10.1073/pnas.90.7.2895 PMC46203 8385349

[31] Stiegler AL Burden SJ Hubbard SR. Crystal structure of the frizzled-like cysteine-rich domain of the receptor tyrosine kinase MuSK. J Mol Biol. (2009) 393:1–9. doi: 10.1016/j.jmb.2009.07.091 19664639 PMC2754272

[32] DeChiara TM Bowen DC Valenzuela DM Simmons MV Poueymirou WT Thomas S. The receptor tyrosine kinase MuSK is required for neuromuscular junction formation *in vivo* . Cell. (1996) 85:501–12. doi: 10.1016/S0092-8674(00)81251-9 8653786

[33] Hubbard SR Gnanasambandan K. Structure and activation of MuSK, a receptor tyrosine kinase central to neuromuscular junction formation. Biochim Biophys Acta. (2013) 1834:2166–9. doi: 10.1016/j.bbapap.2013.02.034 PMC3923368 23467009

[34] Stiegler AL Burden SJ Hubbard SR. Crystal structure of the agrin-responsive immunoglobulin-like domains 1 and 2 of the receptor tyrosine kinase MuSK. J Mol Biol. (2006) 364:424–33. doi: 10.1016/j.jmb.2006.09.019 PMC1752213 17011580

[35] Kim N Stiegler AL Cameron TO Hallock PT Gomez AM Huang JH. Lrp4 is a receptor for Agrin and forms a complex with MuSK. Cell. (2008) 135:334–42. doi: 10.1016/j.cell.2008.10.002 PMC2933840 18848351

[36] Weatherbee SD Anderson KV Niswander LA. LDL-receptor-related protein 4 is crucial for formation of the neuromuscular junction. Development. (2006) 133:4993–5000. doi: 10.1242/dev.02696 17119023

[37] Xie T Xu G Liu Y Quade B Lin W Bai XC. Structural insights into the assembly of the agrin/LRP4/MuSK signaling complex. Proc Natl Acad Sci U S A. (2023) 120:e2300453120. doi: 10.1073/pnas.2300453120 37252960 PMC10266037

[38] Zhang W Coldefy AS Hubbard SR Burden SJ. Agrin binds to the N-terminal region of Lrp4 protein and stimulates association between Lrp4 and the first immunoglobulin-like domain in muscle-specific kinase (MuSK). J Biol Chem. (2011) 286:40624–30. doi: 10.1074/jbc.M111.279307 PMC3220470 21969364

[39] Zong Y Zhang B Gu S Lee K Zhou J Yao G. Structural basis of agrin-LRP4-MuSK signaling. Genes Dev. (2012) 26:247–58. doi: 10.1101/gad.180885.111 PMC3278892 22302937

[40] Bergamin E Hallock PT Burden SJ Hubbard SR. The cytoplasmic adaptor protein Dok7 activates the receptor tyrosine kinase MuSK via dimerization. Mol Cell. (2010) 39:100–9. doi: 10.1016/j.molcel.2010.06.007 PMC2917201 20603078

[41] Xing G Jing H Yu Z Chen P Wang H Xiong WC. Membraneless condensates by Rapsn phase separation as a platform for neuromuscular junction formation. Neuron. (2021) 109:1963–78.e5. doi: 10.1016/j.neuron.2021.04.021 34033754 PMC8217331

[42] Finn AJ Feng G Pendergast AM. Postsynaptic requirement for Abl kinases in assembly of the neuromuscular junction. Nat Neurosci. (2003) 6:717–23. doi: 10.1038/nn1071 12796783

[43] Hallock PT Xu CF Park TJ Neubert TA Curran T Burden SJ. Dok-7 regulates neuromuscular synapse formation by recruiting Crk and Crk-L. Genes Dev. (2010) 24:2451–61. doi: 10.1101/gad.1977710 PMC2964755 21041412

[44] Mittaud P Marangi PA Erb-Vogtli S Fuhrer C. Agrin-induced activation of acetylcholine receptor-bound Src family kinases requires Rapsyn and correlates with acetylcholine receptor clustering. J Biol Chem. (2001) 276:14505–13. doi: 10.1074/jbc.M007024200 11278328

[45] Mohamed AS Rivas-Plata KA Kraas JR Saleh SM Swope SL. Src-class kinases act within the agrin/MuSK pathway to regulate acetylcholine receptor phosphorylation, cytoskeletal anchoring, and clustering. J Neurosci. (2001) 21:3806–18. doi: 10.1523/JNEUROSCI.21-11-03806.2001 PMC6762727 11356869

[46] Weston C Gordon C Teressa G Hod E Ren XD Prives J. Cooperative regulation by Rac and Rho of agrin-induced acetylcholine receptor clustering in muscle cells. J Biol Chem. (2003) 278:6450–5. doi: 10.1074/jbc.M210249200 12473646

[47] Bai Y Guo D Sun X Tang G Liao T Peng Y. Balanced Rac1 activity controls formation and maintenance of neuromuscular acetylcholine receptor clusters. J Cell Sci. (2018) 131(15):jcs215251. doi: 10.1242/jcs.215251 30012833

[48] Bartoli M Ramarao MK Cohen JB. Interactions of the rapsyn RING-H2 domain with dystroglycan. J Biol Chem. (2001) 276:24911–7. doi: 10.1074/jbc.M103258200 11342559

[49] Li L Cao Y Wu H Ye X Zhu Z Xing G. Enzymatic activity of the scaffold protein rapsyn for synapse formation. Neuron. (2016) 92:1007–19. doi: 10.1016/j.neuron.2016.10.023 PMC5366040 27839998

[50] Huh KH Fuhrer C. Clustering of nicotinic acetylcholine receptors: from the neuromuscular junction to interneuronal synapses. Mol Neurobiol. (2002) 25:79–112. doi: 10.1385/MN:25:1:079 11890459

[51] Lee Y Rudell J Ferns M. Rapsyn interacts with the muscle acetylcholine receptor via alpha-helical domains in the alpha, beta, and epsilon subunit intracellular loops. Neuroscience. (2009) 163:222–32. doi: 10.1016/j.neuroscience.2009.05.057 PMC2728176 19482062

[52] Zuber B Unwin N. Structure and superorganization of acetylcholine receptor-rapsyn complexes. Proc Natl Acad Sci U S A. (2013) 110:10622–7. doi: 10.1073/pnas.1301277110 PMC3696831 23754381

[53] Borges LS Ferns M. Agrin-induced phosphorylation of the acetylcholine receptor regulates cytoskeletal anchoring and clustering. J Cell Biol. (2001) 153:1–12. doi: 10.1083/jcb.153.1.1 11285269 PMC2185523

[54] Chen F Qian L Yang ZH Huang Y Ngo ST Ruan NJ. Rapsyn interaction with calpain stabilizes AChR clusters at the neuromuscular junction. Neuron. (2007) 55:247–60. doi: 10.1016/j.neuron.2007.06.031 17640526

[55] Lin W Dominguez B Yang J Aryal P Brandon EP Gage FH. Neurotransmitter acetylcholine negatively regulates neuromuscular synapse formation by a Cdk5-dependent mechanism. Neuron. (2005) 46:569–79. doi: 10.1016/j.neuron.2005.04.002 15944126

[56] Decker ER Dani JA. Calcium permeability of the nicotinic acetylcholine receptor: the single-channel calcium influx is significant. J Neurosci. (1990) 10:3413–20. doi: 10.1523/JNEUROSCI.10-10-03413.1990 PMC6570188 2170596

[57] Engel AG Lambert EH Mulder DM Torres CF Sahashi K Bertorini TE. A newly recognized congenital myasthenic syndrome attributed to a prolonged open time of the acetylcholine-induced ion channel. Ann Neurol. (1982) 11:553–69. doi: 10.1002/ana.410110603 6287911

[58] Ohno K Hutchinson DO Milone M Brengman JM Bouzat C Sine SM. Congenital myasthenic syndrome caused by prolonged acetylcholine receptor channel openings due to a mutation in the M2 domain of the epsilon subunit. Proc Natl Acad Sci U.S.A. (1995) 92:758–62. doi: 10.1073/pnas.92.3.758 PMC42699 7531341

[59] Zhao XT Qian YK Chan AW Madhavan R Peng HB. Regulation of ACh receptor clustering by the tyrosine phosphatase Shp2. Dev Neurobiol. (2007) 67:1789–801. doi: 10.1002/dneu.v67:13 17659592

[60] Zhu D Yang Z Luo Z Luo S Xiong WC Mei L. Muscle-specific receptor tyrosine kinase endocytosis in acetylcholine receptor clustering in response to agrin. J Neurosci. (2008) 28:1688–96. doi: 10.1523/JNEUROSCI.4130-07.2008 PMC6671531 18272689

[61] Gemza A Barresi C Proemer J Hatami J Lazaridis M Herbst R. Internalization of muscle-specific kinase is increased by Agrin and independent of kinase-activity, Lrp4 and dynamin. Front Mol Neurosci. (2022) 15:780659. doi: 10.3389/fnmol.2022.780659 35370548 PMC8965242

[62] Zhang B Liang C Bates R Yin Y Xiong WC Mei L. Wnt proteins regulate acetylcholine receptor clustering in muscle cells. Mol Brain. (2012) 5:7. doi: 10.1186/1756-6606-5-7 22309736 PMC3296622

[63] Yilmaz A Kattamuri C Ozdeslik RN Schmiedel C Mentzer S Schorl C. MuSK is a BMP co-receptor that shapes BMP responses and calcium signaling in muscle cells. Sci Signal. (2016) 9:ra87. doi: 10.1126/scisignal.aaf0890 27601729 PMC5242376

[64] Koneczny I. Update on IgG4-mediated autoimmune diseases: New insights and new family members. Autoimmun Rev. (2020) 19:102646. doi: 10.1016/j.autrev.2020.102646 32801046

[65] Rispens T Huijbers MG. The unique properties of IgG4 and its roles in health and disease. Nat Rev Immunol. (2023) 23:763–78. doi: 10.1038/s41577-023-00871-z PMC10123589 37095254

[66] Huijbers MG Zhang W Klooster R Niks EH Friese MB Straasheijm KR. MuSK IgG4 autoantibodies cause myasthenia gravis by inhibiting binding between MuSK and Lrp4. Proc Natl Acad Sci U.S.A. (2013) 110:20783–8. doi: 10.1073/pnas.1313944110 PMC3870730 24297891

[67] Koneczny I Cossins J Waters P Beeson D Vincent A. MuSK myasthenia gravis IgG4 disrupts the interaction of LRP4 with MuSK but both IgG4 and IgG1-3 can disperse preformed agrin-independent AChR clusters. PLoS One. (2013) 8:e80695. doi: 10.1371/journal.pone.0080695 24244707 PMC3820634

[68] Vidarsson G Dekkers G Rispens T. IgG subclasses and allotypes: from structure to effector functions. Front Immunol. (2014) 5:520. doi: 10.3389/fimmu.2014.00520 25368619 PMC4202688

[69] Schuurman J Perdok GJ Gorter AD Aalberse RC. The inter-heavy chain disulfide bonds of IgG4 are in equilibrium with intra-chain disulfide bonds. Mol Immunol. (2001) 38:1–8. doi: 10.1016/S0161-5890(01)00050-5 11483205

[70] Bloom JW Madanat MS Marriott D Wong T Chan SY. Intrachain disulfide bond in the core hinge region of human IgG4. Protein Sci. (1997) 6:407–15. doi: 10.1002/pro.5560060217 PMC2143633 9041643

[71] Koneczny I Stevens JA De Rosa A Huda S Huijbers MG Saxena A. IgG4 autoantibodies against muscle-specific kinase undergo Fab-arm exchange in myasthenia gravis patients. J Autoimmun. (2017) 77:104–15. doi: 10.1016/j.jaut.2016.11.005 27965060

[72] Young E Lock E Ward DG Cook A Harding S Wallis GL. Estimation of polyclonal IgG4 hybrids in normal human serum. Immunology. (2014) 142:406–13. doi: 10.1111/imm.2014.142.issue-3 PMC4080956 24512211

[73] Huijbers MG Vink AF Niks EH Westhuis RH van Zwet EW de Meel RH. Longitudinal epitope mapping in MuSK myasthenia gravis: implications for disease severity. J Neuroimmunol. (2016) 291:82–8. doi: 10.1016/j.jneuroim.2015.12.016 26857500

[74] Otsuka K Ito M Ohkawara B Masuda A Kawakami Y Sahashi K. Collagen Q and anti-MuSK autoantibody competitively suppress agrin/LRP4/MuSK signaling. Sci Rep. (2015) 5:13928. doi: 10.1038/srep13928 26355076 PMC4564764

[75] Morsch M Reddel SW Ghazanfari N Toyka KV Phillips WD. Muscle specific kinase autoantibodies cause synaptic failure through progressive wastage of postsynaptic acetylcholine receptors. Exp Neurol. (2012) 237:286–95. doi: 10.1016/j.expneurol.2012.06.034 22789393

[76] Viegas S Jacobson L Waters P Cossins J Jacob S Leite MI. Passive and active immunization models of MuSK-Ab positive myasthenia: electrophysiological evidence for pre and postsynaptic defects. Exp Neurol. (2012) 234:506–12. doi: 10.1016/j.expneurol.2012.01.025 22326541

[77] Mori S Kubo S Akiyoshi T Yamada S Miyazaki T Hotta H. Antibodies against muscle-specific kinase impair both presynaptic and postsynaptic functions in a murine model of myasthenia gravis. Am J Pathol. (2012) 180:798–810. doi: 10.1016/j.ajpath.2011.10.031 22142810

[78] Barik A Lu Y Sathyamurthy A Bowman A Shen C Li L. LRP4 is critical for neuromuscular junction maintenance. J Neurosci. (2014) 34:13892–905. doi: 10.1523/JNEUROSCI.1733-14.2014 PMC4198535 25319686

[79] Yumoto N Kim N Burden SJ. Lrp4 is a retrograde signal for presynaptic differentiation at neuromuscular synapses. Nature. (2012) 489:438–42. doi: 10.1038/nature11348 PMC3448831 22854782

[80] Patel V Oh A Voit A Sultatos LG Babu GJ Wilson BA. Altered active zones, vesicle pools, nerve terminal conductivity, and morphology during experimental MuSK myasthenia gravis. PloS One. (2014) 9:e110571. doi: 10.1371/journal.pone.0110571 25438154 PMC4249869

[81] Krejci E Thomine S Boschetti N Legay C Sketelj J Massoulie J. The mammalian gene of acetylcholinesterase-associated collagen. J Biol Chem. (1997) 272:22840–7. doi: 10.1074/jbc.272.36.22840 9278446

[82] Ohno K Brengman J Tsujino A Engel AG. Human endplate acetylcholinesterase deficiency caused by mutations in the collagen-like tail subunit (ColQ) of the asymmetric enzyme. Proc Natl Acad Sci U.S.A. (1998) 95:9654–9. doi: 10.1073/pnas.95.16.9654 PMC21394 9689136

[83] Kawakami Y Ito M Hirayama M Sahashi K Ohkawara B Masuda A. Anti-MuSK autoantibodies block binding of collagen Q to MuSK. Neurology. (2011) 77:1819–26. doi: 10.1212/WNL.0b013e318237f660 PMC3233209 22013178

[84] Cao M Liu WW Maxwell S Huda S Webster R Evoli A. IgG1-3 MuSK antibodies inhibit AChR cluster formation, restored by SHP2 inhibitor, despite normal MuSK, DOK7, or AChR subunit phosphorylation. Neurol Neuroimmunol Neuroinflamm. (2023) 10. doi: 10.1212/NXI.0000000000200147 PMC10427144 37582613

[85] Huijbers MG Vergoossen DL Fillie-Grijpma YE van Es IE Koning MT Slot LM. MuSK myasthenia gravis monoclonal antibodies: Valency dictates pathogenicity. Neurol Neuroimmunol Neuroinflamm. (2019) 6:e547. doi: 10.1212/NXI.0000000000000547 30882021 PMC6410930

[86] Takata K Stathopoulos P Cao M Mane-Damas M Fichtner ML Benotti ES. Characterization of pathogenic monoclonal autoantibodies derived from muscle-specific kinase myasthenia gravis patients. JCI Insight. (2019) 4. doi: 10.1172/jci.insight.127167 PMC6629167 31217355

[87] Vergoossen DLE Plomp JJ Gstottner C Fillie-Grijpma YE Augustinus R Verpalen R. Functional monovalency amplifies the pathogenicity of anti-MuSK IgG4 in myasthenia gravis. Proc Natl Acad Sci U.S.A. (2021) 118(13):e2020635118. doi: 10.1073/pnas.2020635118 33753489 PMC8020787

[88] Rodriguez Cruz PM Cossins J Cheung J Maxwell S Jayawant S Herbst R. Congenital myasthenic syndrome due to mutations in MUSK suggests that the level of MuSK phosphorylation is crucial for governing synaptic structure. Hum Mutat. (2020) 41:619–31. doi: 10.1002/humu.23949 PMC7028094 31765060

[89] Cocanougher BT Liu SW Francescatto L Behura A Anneling M Jackson DG. The severity of MUSK pathogenic variants is predicted by the protein domain they disrupt. HGG Adv. (2024) 5:100288. doi: 10.1016/j.xhgg.2024.100288 38566418 PMC11070630

[90] Promer J Barresi C Herbst R. From phosphorylation to phenotype - Recent key findings on kinase regulation, downstream signaling and disease surrounding the receptor tyrosine kinase MuSK. Cell Signal. (2023) 104:110584. doi: 10.1016/j.cellsig.2022.110584 36608736

[91] Kucukerden M Huda R Tuzun E Yilmaz A Skriapa L Trakas N. MuSK induced experimental autoimmune myasthenia gravis does not require IgG1 antibody to MuSK. J Neuroimmunol. (2016) 295-296:84–92. doi: 10.1016/j.jneuroim.2016.04.003 27235354

[92] Ulusoy C Kim E Tuzun E Huda R Yilmaz V Poulas K. Preferential production of IgG1, IL-4 and IL-10 in MuSK-immunized mice. Clin Immunol. (2014) 151:155–63. doi: 10.1016/j.clim.2014.02.012 24589747

[93] Klooster R Plomp JJ Huijbers MG Niks EH Straasheijm KR Detmers FJ. Muscle-specific kinase myasthenia gravis IgG4 autoantibodies cause severe neuromuscular junction dysfunction in mice. Brain. (2012) 135:1081–101. doi: 10.1093/brain/aws025 22396395

[94] Zisimopoulou P Evangelakou P Tzartos J Lazaridis K Zouvelou V Mantegazza R. A comprehensive analysis of the epidemiology and clinical characteristics of anti-LRP4 in myasthenia gravis. J Autoimmun. (2014) 52:139–45. doi: 10.1016/j.jaut.2013.12.004 24373505

[95] Yasuda M Uzawa A Kuwabara S Suzuki S Akamine H Onishi Y. Clinical features and outcomes of patients with muscle-specific kinase antibody-positive myasthenia gravis in Japan. J Neuroimmunol. (2023) 385:578241. doi: 10.1016/j.jneuroim.2023.578241 37952282

[96] Boldingh MI Maniaol A Brunborg C Dekker L Lipka A Niks EH. Prevalence and clinical aspects of immigrants with myasthenia gravis in northern Europe. Muscle Nerve. (2017) 55:819–27. doi: 10.1002/mus.25408 27641227

[97] Stickler DE Massey JM Sanders DB. MuSK-antibody positive myasthenia gravis: clinical and electrodiagnostic patterns. Clin Neurophysiol. (2005) 116:2065–8. doi: 10.1016/j.clinph.2005.06.003 16043398

[98] Zhang X Yang M Xu J Zhang M Lang B Wang W. Clinical and serological study of myasthenia gravis in HuBei Province, China. J Neurol Neurosurg Psychiatry. (2007) 78:386–90. doi: 10.1136/jnnp.2006.100545 PMC2077769 17088330

[99] Zhang Z Guan Y Han J Li M Shi M Deng H. Regional features of MuSK antibody-positive myasthenia gravis in northeast China. Front Neurol. (2020) 11:516211. doi: 10.3389/fneur.2020.516211 33123066 PMC7566902

[100] Sechi E Deiana GA Puci M Zara P Ortu E Porcu C. Epidemiology of seropositive myasthenia gravis in Sardinia: A population-based study in the district of Sassari. Muscle Nerve. (2024) 69:637–42. doi: 10.1002/mus.28077 38456240

[101] Niks EH Kuks JB Verschuuren JJ. Epidemiology of myasthenia gravis with anti-muscle specific kinase antibodies in The Netherlands. J Neurol Neurosurg Psychiatry. (2007) 78:417–8. doi: 10.1136/jnnp.2006.102517 PMC2077782 17056627

[102] Tsiamalos P Kordas G Kokla A Poulas K Tzartos SJ. Epidemiological and immunological profile of muscle-specific kinase myasthenia gravis in Greece. Eur J Neurol. (2009) 16:925–30. doi: 10.1111/j.1468-1331.2009.02624.x 19374661

[103] Santos E Coutinho E Moreira I Silva AM Lopes D Costa H. Epidemiology of myasthenia gravis in Northern Portugal: Frequency estimates and clinical epidemiological distribution of cases. Muscle Nerve. (2016) 54:413–21. doi: 10.1002/mus.25068 26851892

[104] Westerberg E Punga AR. Epidemiology of myasthenia gravis in Sweden 2006-2016. Brain Behav. (2020) 10:e01819. doi: 10.1002/brb3.v10.11 32869520 PMC7667338

[105] Rosati G. The prevalence of multiple sclerosis in the world: an update. Neurol Sci. (2001) 22:117–39. doi: 10.1007/s100720170011 11603614

[106] Piga M Casula L Perra D Sanna S Floris A Antonelli A. Population-based analysis of hospitalizations in a West-European region revealed major changes in hospital utilization for patients with systemic lupus erythematosus over the period 2001-2012. Lupus. (2016) 25:28–37. doi: 10.1177/0961203315596597 26199283

[107] Steri M Orru V Idda ML Pitzalis M Pala M Zara I. Overexpression of the cytokine BAFF and autoimmunity risk. N Engl J Med. (2017) 376:1615–26. doi: 10.1056/NEJMoa1610528 PMC5605835 28445677

[108] Hong Y Li HF Romi F Skeie GO Gilhus NE. HLA and MuSK-positive myasthenia gravis: A systemic review and meta-analysis. Acta Neurol Scand. (2018) 138:219–26. doi: 10.1111/ane.2018.138.issue-3 29736936

[109] Evoli A Alboini PE Iorio R Damato V Bartoccioni E. Pattern of ocular involvement in myasthenia gravis with MuSK antibodies. J Neurol Neurosurg Psychiatry. (2017) 88:761–3. doi: 10.1136/jnnp-2017-315782 28601810

[110] Rodolico C Parisi D Portaro S Biasini F Sinicropi S Ciranni A. Myasthenia gravis: unusual presentations and diagnostic pitfalls. J Neuromuscul Dis. (2016) 3:413–8. doi: 10.3233/JND-160148 27854225

[111] Zouvelou V Rentzos M Toulas P Evdokimidis I. MRI evidence of early muscle atrophy in MuSK positive myasthenia gravis. J Neuroimaging. (2011) 21:303–5. doi: 10.1111/j.1552-6569.2009.00456.x 20002967

[112] Farrugia ME Kennett RP Newsom-Davis J Hilton-Jones D Vincent A. Single-fiber electromyography in limb and facial muscles in muscle-specific kinase antibody and acetylcholine receptor antibody myasthenia gravis. Muscle Nerve. (2006) 33:568–70. doi: 10.1002/mus.20491 16382443

[113] Kim SW Sunwoo MK Kim SM Shin HY Sunwoo IN. Repetitive nerve stimulation in MuSK-antibody-positive myasthenia gravis. J Clin Neurol. (2017) 13:287–92. doi: 10.3988/jcn.2017.13.3.287 PMC5532326 28516744

[114] Oh SJ Hatanaka Y Hemmi S Young AM Scheufele ML Nations SP. Repetitive nerve stimulation of facial muscles in MuSK antibody-positive myasthenia gravis. Muscle Nerve. (2006) 33:500–4. doi: 10.1002/mus.20498 16392120

[115] Hatanaka Y Hemmi S Morgan MB Scheufele ML Claussen GC Wolfe GI. Nonresponsiveness to anticholinesterase agents in patients with MuSK-antibody-positive MG. Neurology. (2005) 65:1508–9. doi: 10.1212/01.wnl.0000183145.91579.74 16275854

[116] Shin HY Park HJ Lee HE Choi YC Kim SM. Clinical and electrophysiologic responses to acetylcholinesterase inhibitors in muSK-antibody-positive myasthenia gravis: evidence for cholinergic neuromuscular hyperactivity. J Clin Neurol. (2014) 10:119–24. doi: 10.3988/jcn.2014.10.2.119 PMC4017014 24829597

[117] McConville J Farrugia ME Beeson D Kishore U Metcalfe R Newsom-Davis J. Detection and characterization of MuSK antibodies in seronegative myasthenia gravis. Ann Neurol. (2004) 55:580–4. doi: 10.1002/ana.20061 15048899

[118] Matthews I Chen S Hewer R McGrath V Furmaniak J Rees Smith B. Muscle-specific receptor tyrosine kinase autoantibodies–a new immunoprecipitation assay. Clin Chim Acta. (2004) 348:95–9. doi: 10.1016/j.cccn.2004.05.008 15369741

[119] Kwon YN Woodhall M Sung JJ Kim KK Lim YM Kim H. Clinical pitfalls and serological diagnostics of MuSK myasthenia gravis. J Neurol. (2023) 270:1478–86. doi: 10.1007/s00415-022-11458-4 PMC9971039 36396811

[120] Li Z Zhang C Chang T Zhang X Yang H Gao F. A multicentre, prospective, double-blind study comparing the accuracy of autoantibody diagnostic assays in myasthenia gravis: the SCREAM study. Lancet Reg Health West Pac. (2023) 38:100846. doi: 10.1016/j.lanwpc.2023.100846 37554174 PMC10404541

[121] Spagni G Gastaldi M Businaro P Chemkhi Z Carrozza C Mascagna G. Comparison of fixed and live cell-based assay for the detection of AChR and MuSK antibodies in myasthenia gravis. Neurol Neuroimmunol Neuroinflamm. (2023) 10. doi: 10.1212/NXI.0000000000200038 PMC9621337 36270951

[122] Huda S Waters P Woodhall M Leite MI Jacobson L De Rosa A. IgG-specific cell-based assay detects potentially pathogenic MuSK-Abs in seronegative MG. Neurol Neuroimmunol Neuroinflamm. (2017) 4:e357. doi: 10.1212/NXI.0000000000000357 28626780 PMC5459793

[123] Modoni A Mastrorosa A Spagni G Evoli A. Cholinergic hyperactivity in patients with myasthenia gravis with MuSK antibodies: A neurophysiological study. Clin Neurophysiol. (2021) 132:1845–9. doi: 10.1016/j.clinph.2021.04.019 34147009

[124] Chroni E Punga AR. Neurophysiological characteristics of MuSK antibody positive myasthenia gravis mice: focal denervation and hypersensitivity to acetylcholinesterase inhibitors. J Neurol Sci. (2012) 316:150–7. doi: 10.1016/j.jns.2011.12.016 22251934

[125] Morsch M Reddel SW Ghazanfari N Toyka KV Phillips WD. Pyridostigmine but not 3,4-diaminopyridine exacerbates ACh receptor loss and myasthenia induced in mice by muscle-specific kinase autoantibody. J Physiol. (2013) 591:2747–62. doi: 10.1113/tjp.2013.591.issue-10 PMC3678053 23440963

[126] Shapira M Tur-Kaspa I Bosgraaf L Livni N Grant AD Grisaru D. A transcription-activating polymorphism in the ACHE promoter associated with acute sensitivity to anti-acetylcholinesterases. Hum Mol Genet. (2000) 9:1273–81. doi: 10.1093/hmg/9.9.1273 10814709

[127] Evoli A Alboini PE Damato V Iorio R. 3,4-Diaminopyridine may improve myasthenia gravis with MuSK antibodies. Neurology. (2016) 86:1070–1. doi: 10.1212/WNL.0000000000002466 26873957

[128] Skjei KL Lennon VA Kuntz NL. Muscle specific kinase autoimmune myasthenia gravis in children: a case series. Neuromuscul Disord. (2013) 23:874–82. doi: 10.1016/j.nmd.2013.07.010 24012245

[129] Bonanno S Pasanisi MB Frangiamore R Maggi L Antozzi C Andreetta F. Amifampridine phosphate in the treatment of muscle-specific kinase myasthenia gravis: a phase IIb, randomized, double-blind, placebo-controlled, double crossover study. SAGE Open Med. (2018) 6:2050312118819013. doi: 10.1177/2050312118819013 30574306 PMC6299310

[130] Khan MM Lustrino D Silveira WA Wild F Straka T Issop Y. Sympathetic innervation controls homeostasis of neuromuscular junctions in health and disease. Proc Natl Acad Sci U.S.A. (2016) 113:746–50. doi: 10.1073/pnas.1524272113 PMC4725522 26733679

[131] Vanhaesebrouck AE Webster R Maxwell S Rodriguez Cruz PM Cossins J Wickens J. beta2-Adrenergic receptor agonists ameliorate the adverse effect of long-term pyridostigmine on neuromuscular junction structure. Brain. (2019) 142:3713–27. doi: 10.1093/brain/awz322 PMC6892641 31633155

[132] Lee M Beeson D Palace J. Therapeutic strategies for congenital myasthenic syndromes. Ann N Y Acad Sci. (2018) 1412:129–36. doi: 10.1111/nyas.2018.1412.issue-1 29381222

[133] Thompson R Bonne G Missier P Lochmuller H. Targeted therapies for congenital myasthenic syndromes: systematic review and steps towards a treatabolome. Emerg Top Life Sci. (2019) 3:19–37. doi: 10.1042/ETLS20180100 30931400 PMC6436731

[134] Baheerathan A Dorsey R Viegas S. Oral salbutamol for symptomatic treatment in MuSK antibody-positive myasthenia gravis: a single-centre experience. Acta Neurol Belg. (2024). doi: 10.1007/s13760-024-02541-w 38561498

[135] Haran M Schattner A Mate A Starobin D Haran G Shtalrid M. Can a rare form of myasthenia gravis shed additional light on disease mechanisms? Clin Neurol Neurosurg. (2013) 115:562–6. doi: 10.1016/j.clineuro.2012.06.038 22854280

[136] Evoli A Alboini PE Bisonni A Mastrorosa A Bartoccioni E. Management challenges in muscle-specific tyrosine kinase myasthenia gravis. Ann N Y Acad Sci. (2012) 1274:86–91. doi: 10.1111/j.1749-6632.2012.06781.x 23252901

[137] Guptill JT Sanders DB. Update on muscle-specific tyrosine kinase antibody positive myasthenia gravis. Curr Opin Neurol. (2010) 23:530–5. doi: 10.1097/WCO.0b013e32833c0982 20613516

[138] Suh J Goldstein JM Nowak RJ. Clinical characteristics of refractory myasthenia gravis patients. Yale J Biol Med. (2013) 86:255–60. PMC3670444 23766745

[139] Evoli A Bianchi MR Riso R Minicuci GM Batocchi AP Servidei S. Response to therapy in myasthenia gravis with anti-MuSK antibodies. Ann N Y Acad Sci. (2008) 1132:76–83. doi: 10.1196/nyas.2008.1132.issue-1 18567856

[140] Oh SJ. Muscle-specific receptor tyrosine kinase antibody positive myasthenia gravis current status. J Clin Neurol. (2009) 5:53–64. doi: 10.3988/jcn.2009.5.2.53 19587811 PMC2706412

[141] Vesperinas-Castro A Cortes-Vicente E. Rituximab treatment in myasthenia gravis. Front Neurol. (2023) 14:1275533. doi: 10.3389/fneur.2023.1275533 37849836 PMC10577386

[142] Dalakas MC. IgG4-mediated neurologic autoimmunities: understanding the pathogenicity of igG4, ineffectiveness of IVIg, and long-lasting benefits of anti-B cell therapies. Neurol Neuroimmunol Neuroinflamm. (2022) 9. doi: 10.1212/NXI.0000000000001116 PMC8630661 34845096

[143] Khosroshahi A Bloch DB Deshpande V Stone JH. Rituximab therapy leads to rapid decline of serum IgG4 levels and prompt clinical improvement in IgG4-related systemic disease. Arthritis Rheumatol. (2010) 62:1755–62. doi: 10.1002/art.27435 20191576

[144] Carruthers MN Topazian MD Khosroshahi A Witzig TE Wallace ZS Hart PA. Rituximab for IgG4-related disease: a prospective, open-label trial. Ann Rheum Dis. (2015) 74:1171–7. doi: 10.1136/annrheumdis-2014-206605 25667206

[145] Hehir MK Hobson-Webb LD Benatar M Barnett C Silvestri NJ Howard JF Jr.. Rituximab as treatment for anti-MuSK myasthenia gravis: Multicenter blinded prospective review. Neurology. (2017) 89:1069–77. doi: 10.1212/WNL.0000000000004341 28801338

[146] Topakian R Zimprich F Iglseder S Embacher N Guger M Stieglbauer K. High efficacy of rituximab for myasthenia gravis: a comprehensive nationwide study in Austria. J Neurol. (2019) 266:699–706. doi: 10.1007/s00415-019-09191-6 30649616

[147] Diaz-Manera J Martinez-Hernandez E Querol L Klooster R Rojas-Garcia R Suarez-Calvet X. Long-lasting treatment effect of rituximab in MuSK myasthenia. Neurology. (2012) 78:189–93. doi: 10.1212/WNL.0b013e3182407982 22218276

[148] Marino M Basile U Spagni G Napodano C Iorio R Gulli F. Long-lasting rituximab-induced reduction of specific-but not total-IgG4 in MuSK-positive myasthenia gravis. Front Immunol. (2020) 11:613. doi: 10.3389/fimmu.2020.00613 32431692 PMC7214629

[149] Koneczny I Mane-Damas M Zong S De Haas S Huda S van Kruining D. A retrospective multicenter study on clinical and serological parameters in patients with MuSK myasthenia gravis with and without general immunosuppression. Front Immunol. (2024) 15:1325171. doi: 10.3389/fimmu.2024.1325171 38715598 PMC11074957

[150] Stathopoulos P Kumar A Nowak RJ O'Connor KC. Autoantibody-producing plasmablasts after B cell depletion identified in muscle-specific kinase myasthenia gravis. JCI Insight. (2017) 2. doi: 10.1172/jci.insight.94263 PMC5621905 28878127

[151] Willcox HN Newsom-Davis J Calder LR. Cell types required for anti-acetylcholine receptor antibody synthesis by cultured thymocytes and blood lymphocytes in myasthenia gravis. Clin Exp Immunol. (1984) 58:97–106. 6236921 PMC1576978

[152] Yi JS Guptill JT Stathopoulos P Nowak RJ O'Connor KC. B cells in the pathophysiology of myasthenia gravis. Muscle Nerve. (2018) 57:172–84. doi: 10.1002/mus.25973 PMC5767142 28940642

[153] Triplett JD Hardy TA Riminton DS Chu SYK Reddel SW. Association between musk antibody concentrations and the myasthenia gravis composite score in 3 patients: A marker of relapse? Muscle Nerve. (2019) 60:307–11. doi: 10.1002/mus.26609 31177576

[154] Fichtner ML Hoehn KB Ford EE Mane-Damas M Oh S Waters P. Reemergence of pathogenic, autoantibody-producing B cell clones in myasthenia gravis following B cell depletion therapy. Acta Neuropathol Commun. (2022) 10:154. doi: 10.1186/s40478-022-01454-0 36307868 PMC9617453

[155] Anolik JH Barnard J Owen T Zheng B Kemshetti S Looney RJ. Delayed memory B cell recovery in peripheral blood and lymphoid tissue in systemic lupus erythematosus after B cell depletion therapy. Arthritis Rheumatol. (2007) 56:3044–56. doi: 10.1002/art.22810 17763423

[156] Leandro MJ Cambridge G Ehrenstein MR Edwards JC. Reconstitution of peripheral blood B cells after depletion with rituximab in patients with rheumatoid arthritis. Arthritis Rheumatol. (2006) 54:613–20. doi: 10.1002/art.21617 16447239

[157] Leandro MJ. B-cell subpopulations in humans and their differential susceptibility to depletion with anti-CD20 monoclonal antibodies. Arthritis Res Ther. (2013) 15 Suppl 1:S3. doi: 10.1186/ar3908 PMC3624669 23566754

[158] Pyzik M Kozicky LK Gandhi AK Blumberg RS. The therapeutic age of the neonatal Fc receptor. Nat Rev Immunol. (2023) 23:415–32. doi: 10.1038/s41577-022-00821-1 PMC9891766 36726033

[159] Huijbers MG Plomp JJ van Es IE Fillie-Grijpma YE Kamar-Al Majidi S Ulrichts P. Efgartigimod improves muscle weakness in a mouse model for muscle-specific kinase myasthenia gravis. Exp Neurol. (2019) 317:133–43. doi: 10.1016/j.expneurol.2019.03.001 30851266

[160] Howard JF Jr. Bril V Vu T Karam C Peric S Margania T. Safety, efficacy, and tolerability of efgartigimod in patients with generalised myasthenia gravis (ADAPT): a multicentre, randomised, placebo-controlled, phase 3 trial. Lancet Neurol. (2021) 20:526–36. doi: 10.1016/S1474-4422(21)00159-9 34146511

[161] Bril V Druzdz A Grosskreutz J Habib AA Mantegazza R Sacconi S. Safety and efficacy of rozanolixizumab in patients with generalised myasthenia gravis (MycarinG): a randomised, double-blind, placebo-controlled, adaptive phase 3 study. Lancet Neurol. (2023) 22:383–94. doi: 10.1016/S1474-4422(23)00077-7 37059507

[162] Meisel A. Are CAR T cells the answer to myasthenia gravis therapy? Lancet Neurol. (2023) 22:545–6. doi: 10.1016/S1474-4422(23)00211-9 37353270

[163] Habib AA Zhao C Aban I França MC José JG Hörste GM. LUMINESCE, a phase 3 study of satralizumab in generalized myasthenia gravis (gMG): baseline characteristics. Neurology. (2024) 102. doi: 10.1212/WNL.0000000000205247

[164] Yin J Zhao M Xu X Zhang M Xu Z Li Z. A multicenter, randomized, open-label, phase 2 clinical study of telitacicept in adult patients with generalized myasthenia gravis. Eur J Neurol. (2024) 31:e16322. doi: 10.1111/ene.16322 38726639 PMC11235933

[165] Hewett K Sanders DB Grove RA Broderick CL Rudo TJ Bassiri A. Randomized study of adjunctive belimumab in participants with generalized myasthenia gravis. Neurology. (2018) 90:e1425–e34. doi: 10.1212/WNL.0000000000005323 PMC5902787 29661905

[166] Schneider-Gold C Reinacher-Schick A Ellrichmann G Gold R. Bortezomib in severe MuSK-antibody positive myasthenia gravis: first clinical experience. Ther Adv Neurol Disord. (2017) 10:339–41. doi: 10.1177/1756285617721093 PMC5607927 28966662

[167] Granit V Benatar M Kurtoglu M Miljkovic MD Chahin N Sahagian G. Safety and clinical activity of autologous RNA chimeric antigen receptor T-cell therapy in myasthenia gravis (MG-001): a prospective, multicentre, open-label, non-randomised phase 1b/2a study. Lancet Neurol. (2023) 22:578–90. doi: 10.1016/S1474-4422(23)00194-1 PMC10416207 37353278

[168] Oh S Mao X Manfredo-Vieira S Lee J Patel D Choi EJ. Precision targeting of autoantigen-specific B cells in muscle-specific tyrosine kinase myasthenia gravis with chimeric autoantibody receptor T cells. Nat Biotechnol. (2023) 41:1229–38. doi: 10.1038/s41587-022-01637-z PMC10354218 36658341

[169] Oury J Gamallo-Lana B Santana L Steyaert C Vergoossen DLE Mar AC. Agonist antibody to MuSK protects mice from MuSK myasthenia gravis. Proc Natl Acad Sci U.S.A. (2024) 121:e2408324121. doi: 10.1073/pnas.2408324121 39288173 PMC11441477

[170] Albazli K Kaminski HJ Howard JF Jr. Complement inhibitor therapy for myasthenia gravis. Front Immunol. (2020) 11:917. doi: 10.3389/fimmu.2020.00917 32582144 PMC7283905

[171] Howard JF Jr. Bresch S Genge A Hewamadduma C Hinton J Hussain Y. Safety and efficacy of zilucoplan in patients with generalised myasthenia gravis (RAISE): a randomised, double-blind, placebo-controlled, phase 3 study. Lancet Neurol. (2023) 22:395–406. doi: 10.1016/S1474-4422(23)00080-7 37059508

[172] Vu T Meisel A Mantegazza R Annane D Katsuno M Aguzzi R. Terminal complement inhibitor ravulizumab in generalized myasthenia gravis. NEJM Evid. (2022) 1:EVIDoa2100066. doi: 10.1056/EVIDoa2100066 38319212

[173] Pallaver F Riviera AP Piffer S Ricciardi R Roni R Orrico D. Change in myasthenia gravis epidemiology in Trento, Italy, after twenty years. Neuroepidemiology. (2011) 36:282–7. doi: 10.1159/000328863 21757957

[174] Zieda A Ravina K Glazere I Pelcere L Naudina MS Liepina L. A nationwide epidemiological study of myasthenia gravis in Latvia. Eur J Neurol. (2018) 25:519–26. doi: 10.1111/ene.2018.25.issue-3 29194859

